# Candidate Gene Sequencing of *LHX2*, *HESX1*, and *SOX2* in a Large Schizencephaly Cohort

**DOI:** 10.1002/ajmg.a.33684

**Published:** 2010-10-11

**Authors:** Cecilia Mellado, Annapurna Poduri, Danielle Gleason, Princess C Elhosary, Brenda J Barry, Jennifer N Partlow, Bernard S Chang, Gary M Shaw, A James Barkovich, Christopher A Walsh

**Affiliations:** 1 Department of Neurology, Children's Hospital Boston and Harvard Medical SchoolBoston, Massachusetts; 2 Department of Pediatrics, Pontificia Universidad Católica de ChileSantiago, Chile; 3 Division of Genetics, The Manton Center for Orphan Disease Research, Children's Hospital BostonBoston, Massachusetts; 4 Department of Neurology, Beth Israel Deaconess Medical Center and Harvard Medical SchoolBoston, Massachusetts; 5 Department of Pediatrics, Stanford UniversitySan Francisco, California; 6 Division of Pediatric Neuroradiology, Department of Neuroradiology, University of CaliforniaSan Francisco, California; 7 Howard Hughes Medical InstituteBoston, Massachusetts

**Keywords:** schizencephaly, septo-optic dysplasia, *LHX1*, *HESX1*, *SOX2*

## Abstract

Schizencephaly is a malformation of cortical development characterized by gray matter-lined clefts in the cerebral cortex and a range of neurological presentations. In some cases, there are features of septo-optic dysplasia concurrently with schizencephaly. The etiologies of both schizencephaly and septo-optic dysplasia are thought to be heterogeneous, but there is evidence that at least some cases have genetic origin. We hypothesized that these disorders may be caused by mutations in three candidate genes: *LHX2*, a gene with an important cortical patterning role, and *HESX1* and *SOX2*, genes that have been associated with septo-optic dysplasia. We sequenced a large cohort of patients with schizencephaly, some with features of septo-optic dysplasia, for mutations in these genes. No pathogenic mutations were observed, suggesting that other genes or non-genetic factors influencing genes critical to brain development must be responsible for schizencephaly. © 2010 Wiley-Liss, Inc.

## INTRODUCTION

Schizencephaly is an uncommon malformation of cortical development with an estimated prevalence in the United States of 1.54 per 100,000 individuals [Curry et al., 2005]. Patients with schizencephaly present with a broad range of neurological symptoms, including developmental delay, seizures, and hemiparesis or quadriparesis. Neuroimaging studies show unilateral or bilateral full thickness gray matter-lined clefts of the cerebral hemispheres. The two sides of a schizencephalic cleft can appear fused with a pial-ependymal seam (termed “closed lip”), or they can be separated by cerebrospinal fluid (“open lip”) [Yakovlev and Wadsworth, 1946a,b]. Such clefts are most often found in the perisylvian region, and the surrounding cortex is typically polymicrogyric [Barkovich and Norman, 1988; Barkovich and Kjos, 1992; Granata et al., 1996; Hayashi et al., 2002].

The suspected causes of schizencephaly are heterogeneous and remain poorly understood. Both genetic and non-genetic etiologies have been postulated [Bubis and Landau, 1964; Komarniski et al., 1990; Hehr et al., 2010]. There are few reported associations with chromosomal aneuploidy, single gene defects, and distinct syndromes. Schizencephaly is usually simplex, but familial occurrence has been reported indicating one or more genetic factors for schizencephaly [Robinson 1991; Hosley et al., 1992; Hilburger et al., 1993; Tietjen et al., 2005].

A role for *EMX2* was initially suggested in reports of multiplex and simplex cases [Capra et al., 1996; Faiella et al., 1997; Granata et al., 1997], but more recent studies have not demonstrated a major role for *EMX2* in schizencephaly [Tietjen et al., 2007; Merello et al., 2008; Hehr et al., 2010]. *LHX2*, another gene expressed in the developing forebrain, specifies cortical fate in the hippocampus and neocortex [Hebert and Fishell, 2008] and has been hypothesized as a potential candidate gene for schizencephaly. *Lhx2* knockout mice have a striking phenotype with an essentially absent neocortex [Monuki and Walsh, 2001; Mangale et al., 2008].

Schizencephaly is often associated with other CNS anomalies, including hydrocephalus, arachnoid cysts, and partial or complete absence of the septum pellucidum (ASP). In fact, up to 70% of cases of schizencephaly are associated with absent or abnormal septum pellucidum, and up to one-fourth of schizencephaly cases fall into the spectrum of septo-optic dysplasia (SOD) [Barkovich and Norman, 1988; Barkovich et al., 1989; Barkovich, 2005]. Conversely, nearly half of the patients reported with SOD have schizencephaly [Barkovich et al., 1989; Lau et al., 1993].

The coexistence of SOD and schizencephaly suggests potential common etiologies for both phenotypes. The majority of patients with SOD are simplex, and several etiologies have been postulated, including genetic abnormalities, viral infections, and vascular events. Some multiplex cases have been reported, supporting the role of genetics underlying SOD [Benner et al., 1990; Wales and Quarrell, 1996; Kelberman and Dattani, 2007]. Both homozygous and heterozygous missense mutations in *HESX1* have been reported in association with SOD [Dattani et al., 1998; Thomas et al., 2001]. Mutations in *HESX1* have also been reported in patients with ectopic posterior pituitary gland [Brickman et al., 2001] and in a patient with periventricular heterotopia and ectopic posterior pituitary gland [Mitchell et al., 2002]. The *Hesx1* homeobox gene is expressed early in development in the mouse forebrain, and there is evidence for an important role for *Hesx1/HESX1* in the development of mouse and human forebrain and pituitary gland [Dattani et al., 1998]. Given the evidence for a role of *HESX1* in SOD and the frequent association between SOD and schizencephaly, we hypothesized that *HESX1* mutations might be responsible for some proportion of simplex cases of schizencephaly.

The report of a patient with SOD and schizencephaly with a heterozygous mutation in *SOX2* (c.389G > C, p.G130A) [Kelberman et al., 2006] makes this another attractive candidate gene for schizencephaly. *SOX2*, expressed in early neuroepithelial progenitors, is a transcription factor implicated in the development of the cerebral cortex, pituitary gland, eye, and inner ear.

Despite the strong suspicion of a genetic etiology for schizencephaly, the majority of cases remain unexplained. Based on the coexistence of schizencephaly and SOD, we hypothesized that mutations in genes previously shown to be mutated in patients with SOD can also cause schizencephaly, particularly in those patients with features of SOD but perhaps also in those without SOD. Given the associations described above, we evaluated 97 cases of schizencephaly, including 13 with both schizencephaly and features of SOD, to screen for mutations in *LHX2*, *HESX1*, and *SOX2*.

## SUBJECTS AND METHODS

### Subjects

This study was conducted with the approval of the Institutional Review Board of Children's Hospital Boston and the Beth Israel Deaconess Medical Center. Subjects with schizencephaly were identified from a large research database of brain malformation cases (Walsh Laboratory) and from the California Birth Defects Monitoring Program (CBDMP). In the cases ascertained from the Walsh Laboratory research database, informed consent was obtained from the patients or their parents/guardians. Data and DNA from the CBDMP were de-identified and made available for inclusion in this study. We reviewed the medical records and MRIs (magnetic resonance imaging) of all potential cases when these data were available. We classified schizencephaly with regard to open versus closed lip and to laterality. In patients with schizencephaly and features consistent with SOD, we performed a more detailed analysis of the MRIs, including SOD-related features, and associated clinical features.

### Genetic Analysis

Blood was collected by venipuncture from patients diagnosed with schizencephaly. Genomic DNA was extracted from peripheral blood lymphocytes using standard techniques. Primers were designed for sequencing of *LHX2*, *HESX1*, and *SOX2*; we sequenced all exons and exon–intron junctions in the forward and reverse directions (Polymorphic DNA Technologies, Alameda, CA). Sequencing data were analyzed using Variant Report and DNAStar software. When polymorphisms were observed, we interpreted them using the UCSC Genome Browser 2006 Assembly (http://www.genome.ucsc.edu). Based on our initial observations, we designed forward and reverse primers for exon 3 of *LHX2* using Primer 3 software, and we screened 574 control chromosomes from a panel of 287 Caucasian control samples.

## RESULTS

### Clinical Description of Schizencephaly Patients

**FIG. 1:**
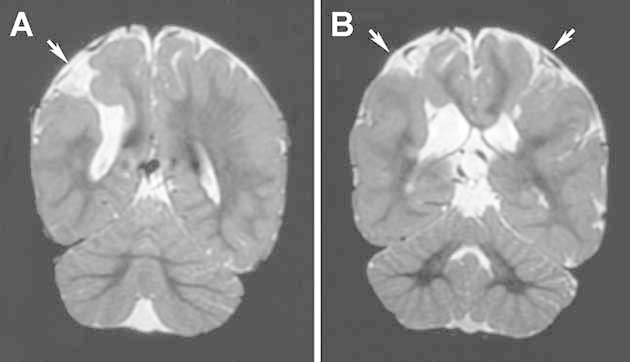
Coronal T2 MRI imaging from two patients demonstrates unilateral, right-sided open lip schizencephaly (**A**) and bilateral schizencephaly with closed lip on the right and open lip on the left (**B**) (arrows).

We analyzed the available clinical and MRI data from 97 patients identified from the Walsh Laboratory research database (n = 39) and the CBDMP (n = 58). We confirmed the diagnosis of schizencephaly when possible by review of MRI data and/or medical records. Representative patients of open and closed lip schizencephaly are shown in Figure 1.

For the CBDMP patients, while only information about the schizencephaly type was available to us, this population has been well described in the past [Curry et al., 2005].

#### Schizencephaly type

Of the 97 patients included in our analysis, there was sufficient information about the laterality and lip type to be able to classify the schizencephaly in 75. Fourteen had unilateral open lip schizencephaly, 13 unilateral closed lip, 20 bilateral open lip, and four bilateral closed lip, as shown in Table I. Five patients had a combination of open and closed lip schizencephaly, one unilateral (two clefts in the same hemisphere) and four bilateral. Nineteen patients had unknown lip type, but information about laterality was available (12 unilateral and seven bilateral).

**TABLE I:** Characterization of Schizencephaly in 75 Cases

Schizencephaly laterality	Lip type				

	Open lip	Closed lip	Open and closed lip	Unknown lip	Total (%)
Unilateral	14	13	1	12	40 (53)
Bilateral	20	4	4	7	35 (47)
Total (%)	34 (45)	17 (23)	5 (7)	19 (25)	75 (100)

#### Patients with Schizencephaly and SOD

We observed 14 patients in which there was schizencephaly with coexistent SOD on the basis of neuroimaging features, such as ASP, optic nerve hypoplasia, and pituitary gland abnormalities, detailed in Table II. In this subset of patients, eight were male and six female; the age at ascertainment ranged from 22 days to 37 years.

**TABLE II:** Description of Patients With Schizencephaly and SOD-Related Imaging Features

Case	Sex	Age	Schizencephaly type	Features consistent with SOD	Other brain imaging findings	Clinical features
1	M	22 day	Left closed lip	Ectopic posterior pituitary lobe	Possible focal cortical dysplasia, left temporal lobe	Macrocephaly
					Ventriculomegaly	
2	M	1 month	Bilateral	Thin optic nerves (autopsy)	Immature gyral pattern	Panhypopituitarism
			Open lip on right	Hypoplastic pituitary	Absent splenium of corpus callosum	Seizures
			Closed lip on left		Ventriculomegaly	Died, 1-month-old
3	F	2 years, 5 months	Bilateral	Hypoplastic pituitary, absent posterior pituitary	Agenesis of the corpus callosum	Microcephaly
			Four open lip clefts		Reduced white matter volume	Developmental delay
					Delayed myelination	Visually impairment
					Hypoplastic brain stem	
4	M	8 months	Right open lip	Hypoplastic pituitary	Generalized polymicrogyria	Hypertelorism
					Agenesis of the corpus callosum	Developmental delay
					Reduced white matter volume	Ventriculo-peritoneal shunt
					Delayed myelination	
					Ventriculomegaly	
5	M	2 years	Bilateral closed lip	Bilateral optic nerve hypoplasia	None	Pendular nystagmus
						Left exotropia
						Spastic quadriplegia
6	F	27 years	Right open lip	Absent septum pellucidum	Left hemisphere polymicrogyria	Seizures
					Thin posterior body of corpus callosum	Developmental delay
					Reduced white matter volume	Left spastic hemiparesis
					Right-sided ventriculomegaly	Choreoathetosis in the left hand
						Dysarthric speech
7	M	20 years	Right open lip	Absent septum pellucidum	Left hemisphere cortical dysplasia	Seizures
					Thin posterior body of corpus callosum	Developmental delay
					Reduced white matter volume	Left spastic hemiparesis
					Right-sided ventriculomegaly	Choreoathetosis in the left hand
						Dysarthric speech
8	M	21 years	Right closed lip	Absent septum pellucidum	Left hemisphere cortical dysplasia	Seizures
					Thin posterior body of the corpus callosum	Developmental delay
					Reduced white matter volume	Left spastic hemiparesis
					Right-sided ventriculomegaly	Choreoathetosis in the left hand
						Dysarthric speech
9	M	2 years	Bilateral open lip	Absent septum pellucidum	Ventricular heterotopia contiguous with clefts	Developmental delay
				Bilateral optic nerve hypoplasia	Dysmorphic (thick, foreshortened) corpus callosum	Spastic quadriparesis
					Reduced white matter volume	
10	F	3 years	Right closed lip	Absent septum pellucidum	Reduced white matter volume	Developmental delay
						Left hemiparesis
11	F	2 years	Right open lip	Absent septum pellucidum	Polymicrogyria	Developmental delay
				Bilateral optic nerve hypoplasia and hypoplasia of optic chiasm	Reduced white matter volume	Left hemiparesis
					Calcifications in ventricular walls	
					Small right cerebral peduncle, pons, and medullary pyramid	
12	F	37 years	Right closed lip	Absent septum pellucidum	Partially fused forniceal columns	Asymptomatic
				Bilateral optic nerve hypoplasia		
13	M	2 years	Bilateral closed lip	Absent septum pellucidum	Hypoplastic corpus callosum	Microcephaly
				Bilateral optic nerve hypoplasia and hypoplasia of optic chiasm	Ventriculomegaly	Developmental delay
				Absent posterior pituitary	Moderately diminished olfactory system	Nystagmus
						Feeding difficulties
						Hypotonia
						Two sisters with schizencephaly and SOD
14	F	4 years	Bilateral	Hypoplastic septum pellucidum	Not available	Microcephaly
						Developmental delay
						Nystagmus
						Feeding difficulties
						Hypotonia

The MRI results for this group demonstrated a range of SOD-related abnormalities. Nine patients had a single SOD-related feature: five had an abnormality of the septum pellucidum (Patients 6, 7, 8, 10, and 14), three had pituitary abnormalities (Patients 1, 3, and 4), and one had bilateral optic nerve hypoplasia (Patient 5). Notably, Patient 3 also had visual impairment, suggesting involvement of the optic pathway, though this was not evident on imaging. Four patients had two SOD-related imaging findings: Patient 2 had thin optic nerves and hypoplastic pituitary, and three patients had ASP plus hypoplasia of the optic nerves or pathway (Cases 9, 11, and 12). Patient 13, had three SOD-related imaging features: ASP, bilateral optic nerve and chiasm hypoplasia, and absent posterior pituitary.

All but patients with schizencephaly and SOD had additional MRI abnormalities, including ventriculomegaly, agenesis or abnormality of the corpus callosum, diminished white matter volume and/or delayed myelination, and polymicrogyria. Some other features occurred in only single patients and are listed in Table II. The clinical features of this group included features classically attributed to schizencephaly (developmental delay, seizures, hemiparesis, and quadriparesis). There was a paucity of SOD-related clinical features reported: only one patient had pituitary dysfunction (Patient 2), and only one had visual impairment (Patient 3).

### Interpretation of candidate gene sequencing

Analysis of sequencing of the *LHX2*, *HESX1*, and *SOX2* exons and exon–intron junctions in 97 cases of schizencephaly did not reveal mutations that were likely to be pathogenic. We observed several polymorphisms, some published and some not previously reported (Table III).

**TABLE III:** Sequence Changes in *LHX2*, *HESX1*, and *SOX2* in Schizencephaly Cases

Gene	Exon	Chromosome position UCSC (hg18, March 2006)	SNP Id (dbSNP129)	Variant	Frequency (%)	Interpretation
*LHX2*	Exon 1	Chr9: 125,814,336	rs7868184	c.-113G > T	4/97 (4)	5′-UTR
	Exon 3	Chr9: 125,817,622	n/a	c.724G > A	1/97 (1)	p.A242T, conserved^a^
	Intron 3	Chr9: 125,823,178	n/a	c.728–21G > T	5/97 (5)	Intronic, not conserved
	Exon 4	Chr9: 125,823,236	rs61734362	c.765C > T	1/97 (1)	p.D255D
	Exon 4	Chr9: 125,823,254	rs1042486	c.783G > C	46/97 (45)	p.P261P
	Exon 5	Chr9: 125,834,603	n/a	c.1017C > G	1/97 (1)	p.A339A
*HESX1*	Exon 3	Chr3: 57,207,544	rs9878928	c.374A > G	3/97 (3)	N125S
*SOX2*	Exon 1	Chr3: 182,913,818	n/a	c.*22 G > A	2/97 (2)	3′-UTR, not conserved

n/a, not applicable.

a 3 bp from rs71801713, a large insertion/deletion polymorphism.

#### LHX2

In a single case of schizencephaly, we observed a heterozygous c.724G > A change, resulting in p.Ala242Thr. Though this is a conserved alanine residue, it is located only 3 bp from a large insertion/deletion polymorphism (rs71801713 at chr9:125,817,625–125,823,197) and does not fall in the region of any of the well characterized functional LHX2 protein domains [http://www.uniprot.org]. We screened 287 Caucasian controls (574 chromosomes) and did not find the c.724G > A change, suggesting that it is not a common polymorphism.

We observed three previously reported SNPs: rs7868184 in exon 1 (n = 4, three heterozygous, one homozygous), rs61734362 in exon 4 (n = 1, heterozygous), and rs1042486 in exon 4 (n = 46, 35 heterozygous, 11 homozygous). The high frequency of rs1042486 in our group of cases is consistent with the reported rates of 42–52% in Caucasian and Asian samples (HapMap-CEU and HapMap-CHB and JPT; http://hapmap.ncbi.nlm.nih.gov). Five patients had a G > T heterozygous change in intron 3; the native G is not highly conserved across species, but the polymorphism occurs in non-coding sequence. One patient had a synonymous change in exon 5, c.1017C > G.

#### HESX1

In three patients with schizencephaly, including one with SOD features (Patient 3) we observed an apparent heterozygous missense mutation in *HESX1*, c.374A > G (p.Asn125Ser). However, since this polymorphism has been observed to occur in 35% of a large, Sub-Saharan African control sample (HapMap-YRI) (http://hapmap.ncbi.nlm.nih.gov) and has been published in dbSNP as rs9878928, we concluded that it was not pathogenic.

#### SOX2

We observed one heterozygous change in *SOX2*: c.*22 G > A (exon 1 + 22 bp) in two patients, including one with SOD features (Case 12). Though not yet published in dbSNP, this polymorphism, occurring in a non-coding nucleotide that is not highly conserved, has been reported in unaffected controls [Fantes et al., 2003; Bakrania et al., 2007].

## DISCUSSION

Though schizencephaly is a heterogeneous condition, we strongly suspect that some cases have an underlying genetic etiology. Nonetheless, there are to date no genes identified that play a major role in this condition. We hypothesized that mutations in *LHX2* might give rise to schizencephaly given the important role of *LHX2* in cortical patterning. Because of the overlap sometimes observed between schizencephaly and SOD, we also hypothesized that genes associated with SOD—*HESX1* and *SOX2*—might also cause some cases of schizencephaly.

We observed several polymorphisms in *LHX2*, including published SNPs. One *LHX2* sequence variation, c.724G > A, had not been previously reported. Though it is synonymous, this does not occur in any of the functionally important LHX2 domains and is adjacent to a large known polymorphism. Thus, we concluded that this change represents a benign though uncommon polymorphism. We observed only one polymorphism in *HESX1*; even though it represented a non-synonymous variation in the amino acid sequence, it is a previously reported common polymorphism (http://hapmap.ncbi.nlm.nih.gov). Similarly, we observed a polymorphism in the 3′-UTR of *SOX2* in a single case that we concluded was not pathogenic [Fantes et al., 2003; Bakrania et al., 2007].

From a total of 97 cases of schizencephaly, there were 14 with concurrent features suggestive of SOD, including ASP, optic nerve hypoplasia, and pituitary gland abnormalities. As this is a retrospective study and full records were not available from all cases, it is difficult to estimate accurately the true coincidence of SOD among all studied cases of schizencephaly. Our findings in a large but heterogeneous group of subjects with schizencephaly, a subset of whom had features of SOD, indicate that these candidate genes do not account for a significant fraction of schizencephaly or schizencephaly + SOD cases.

It is clear from familial studies that at least some occurrences of schizencephaly have a genetic etiology, but the extent to which genetic causes can explain these conditions remains unknown. The observation of schizencephaly in combination with some or all of the features of SOD in some patients may be secondary to concomitant injury to multiple structures in the developing brain. It is plausible that some mutations may cause schizencephaly by increasing the susceptibility of the developing brain to environmental events. Additional studies will be needed to better define the genetic and non-genetic factors that contribute to schizencephaly.
